# T cell receptor gene repertoire profiles in subgroups of patients with chronic lymphocytic leukemia bearing distinct genomic aberrations

**DOI:** 10.3389/fonc.2023.1097942

**Published:** 2023-02-01

**Authors:** Elisavet Vlachonikola, Nikolaos Pechlivanis, Georgios Karakatsoulis, Electra Sofou, Glykeria Gkoliou, Sabine Jeromin, Niki Stavroyianni, Pamela Ranghetti, Lydia Scarfo, Cecilia Österholm, Larry Mansouri, Sofia Notopoulou, Alexandra Siorenta, Achilles Anagnostopoulos, Paolo Ghia, Claudia Haferlach, Richard Rosenquist, Fotis Psomopoulos, Anastasia Kouvatsi, Panagiotis Baliakas, Kostas Stamatopoulos, Anastasia Chatzidimitriou

**Affiliations:** ^1^ Institute of Applied Biosciences, Centre for Research and Technology Hellas, Thessaloniki, Greece; ^2^ Department of Genetics, Development and Molecular Biology, School of Biology, Aristotle, University of Thessaloniki, Thessaloniki, Greece; ^3^ Department of Mathematics, School of Sciences, University of Ioannina, Ioannina, Greece; ^4^ Laboratory of Biological Chemistry, School of Medicine, Aristotle University of Thessaloniki, Thessaloniki, Greece; ^5^ Department of Medicine, Democritus University of Thrace, Alexandroupolis, Greece; ^6^ MLL - Munich Leukemia Laboratory, Munich, Germany; ^7^ Hematology Department and Hematopoietic Cell Transplantation (HCT) Unit, G. Papanicolaou Hospital, Thessaloniki, Greece; ^8^ Division of Experimental Oncology, Università Vita-Salute San Raffaele and Istituto di Ricovero e Cura a Carattere Scientifico (IRCCS), Ospedale San Raffaele, Milan, Italy; ^9^ Department of Molecular Medicine and Surgery, Karolinska Institutet, Stockholm, Sweden; ^10^ Immunology Department and National Tissue Typing Center, General Hospital of Athens “G. Gennimatas”, Athens, Greece; ^11^ Clinical Genetics, Karolinska University Hospital, Solna, Sweden; ^12^ Department of Immunology, Genetics and Pathology, Science for Life Laboratory, Uppsala University, Uppsala, Sweden

**Keywords:** T cell receptor (TR) gene repertoire, chronic lymphocytic leukemia (CLL), recurrent genomic aberrations, neoepitopes, T cell based immunotherapies

## Abstract

**Background:**

Microenvironmental interactions of the malignant clone with T cells are critical throughout the natural history of chronic lymphocytic leukemia (CLL). Indeed, clonal expansions of T cells and shared clonotypes exist between different CLL patients, strongly implying clonal selection by antigens. Moreover, immunogenic neoepitopes have been isolated from the clonotypic B cell receptor immunoglobulin sequences, offering a rationale for immunotherapeutic approaches. Here, we interrogated the T cell receptor (TR) gene repertoire of CLL patients with different genomic aberration profiles aiming to identify unique signatures that would point towards an additional source of immunogenic neoepitopes for T cells.

**Experimental design:**

TR gene repertoire profiling using next generation sequencing in groups of patients with CLL carrying one of the following copy-number aberrations (CNAs): del(11q), del(17p), del(13q), trisomy 12, or gene mutations in *TP53* or *NOTCH1*.

**Results:**

Oligoclonal expansions were found in all patients with distinct recurrent genomic aberrations; these were more pronounced in cases bearing CNAs, particularly trisomy 12, rather than gene mutations. Shared clonotypes were found both within and across groups, which appeared to be CLL-biased based on extensive comparisons against TR databases from various entities. Moreover, *in silico* analysis identified TR clonotypes with high binding affinity to neoepitopes predicted to arise from *TP53* and *NOTCH1* mutations.

**Conclusions:**

Distinct TR repertoire profiles were identified in groups of patients with CLL bearing different genomic aberrations, alluding to distinct selection processes. Abnormal protein expression and gene dosage effects associated with recurrent genomic aberrations likely represent a relevant source of CLL-specific selecting antigens.

## Introduction

In recent years, novel agents targeting critical processes and pathways such as microenvironmental interactions (e.g. BTK inhibitors) and apoptotic cell death (e.g. BCL-2 inhibitors) have revolutionized the management of chronic lymphocytic leukemia (CLL), leading to high overall response rates (often exceeding 90%) and superior progression-free and, at least in some trials, overall survival compared to classic chemoimmunotherapy (1). Nevertheless, relapses still occur and CLL remains an incurable malignancy, highlighting the need for alternative therapeutic approaches aiming at more effective disease control (2). Immunotherapy that engages immune responses against targets on the malignant cells could, at least in principle, represent such an approach also for CLL. However, despite initial promising results, it has become apparent that not all patients with CLL benefit from (different forms of) immunotherapy, highlighting the need for further research into the underlying mechanisms, particularly focusing on T cells (3, 4).

Multiple lines of evidence support that T cells present in the tumor microenvironment (TME) are implicated in the natural history of CLL, albeit in rather contrasting ways (5). On the one hand, T cells provide trophic signals for CLL cell survival. This is evidenced by the fact that transfer of autologous activated T cells is required for the successful engraftment of CLL cells in murine models; and that efficient proliferation of CLL cells was observed after the engraftment of CD4^+^ T cells bearing a T cell receptor (TR) with CLL-unrelated specificity (6–8). On the other hand, CLL cells induce various changes in the T cell compartment, leading to functional exhaustion as a consequence of persistent antigenic stimulation, which underlies tumor evasion and immune suppression (9–12).

Molecular studies by us and others revealed skewing of the TR repertoire and clonal expansions of T cells in CLL, supporting antigen drive (13–15). Moreover, different patients were found to share TR clonotypes, many of which were ‘CLL-biased’ i.e. not present in other settings (16, 17). Altogether, these findings strongly imply ongoing antigenic triggering in a CLL-specific context (16, 17). The cognate antigens remain to be determined and could arguably be those selecting the malignant clone or unrelated ones, including tumor-derived antigens.

Particularly regarding the latter, we have previously demonstrated that immunogenic epitopes can be isolated from the heavy complementarity-determining region 3 (VH CDR3) sequence of both human and murine CLL bearing stereotyped [i.e. (quasi)identical] B cell receptors (BcR), efficiently processed and presented by CLL cells and effectively recognized by specific T cells (18). Of note, immunization of Eμ-TCL1 CLL mice model reduced leukemia development and increased overall survival of the animals (18). Subsequently, we provided evidence that the targeted somatic hypermutation operating on the BcR immunoglobulin (BcR IG) repertoire may produce idiotypic targets for the cognate T cells (19).

Taken together, the clonotypic BcR IG expressed by CLL cells can be envisioned as a source of neoepitopes selecting T cells in the TME. That said, recurrent genomic aberrations associated with distinct abnormal expression profiles could represent an alternative, non-mutually exclusive, pool of such potent immunogenic neoepitopes. Judging from a great variety of other malignancies shown to harbor reactive T cells against epitopes arisen from recurrent genomic aberrations, this may prove clinically relevant, especially if one considers the promising results of immunotherapy in e.g. patients with melanoma or lung cancer exhibiting a high mutational load (20–22).

On these grounds, here we interrogated the TR gene repertoire in groups of patients with CLL carrying specific single genomic aberrations, one of del(11q), del(17p), del(13q), trisomy 12, *TP53* or *NOTCH1* mutations. We report oligoclonality in all groups of patients with distinct recurrent genomic aberrations, albeit more pronounced in cases bearing copy number aberrations (CNAs), particularly trisomy 12. This, combined with the existence of group-specific clonotypes, suggest that abnormal protein expression and gene dosage effects can lead to the emergence of CLL-specific selecting epitopes that likely restrict the TR gene repertoire.

## Materials and methods

### Patient group

The study cohort included 44 patients with CLL from 4 different centers in Thessaloniki, Greece; Milan, Italy; Munich, Germany; and Stockholm, Sweden. The local Ethics Review Committees approved the study, while written informed consent was obtained from all study participants, in accordance with the Declaration of Helsinki.

Patients were selected on the basis of carrying one of the following genomic aberrations: del(11q) (n=10), del(13q) (n=7), trisomy 12 (n=17), *NOTCH1* mutation (n=5) or *TP53* mutation (n=5). The possible confounding effects of multiple aberrations were minimized, as we previously established that the analyzed patients carried only one of the aforementioned aberrations through comprehensive genomic characterization (including FISH, SNP arrays, gene panels and WES; for methodological details, see **Supplementary Methods**). Demographic and clinicobiological characteristics are provided in **Supplementary Table S1**. In all patients, samples were collected before the initiation of any treatment and there was no evidence of infection (either signs or symptoms), or recent vaccination at sampling that could bias the results.

### Next-generation sequencing: Library preparation, analysis and interpretation

T cell receptor beta chain (TRB) gene rearrangements were amplified on either genomic DNA or complementary DNA (cDNA) isolated from peripheral blood mononuclear cells (PBMCs). In all samples, the starting absolute PBMC count was set at 5x10^6^ cells in order to determine actual repertoire diversity while avoiding to over-amplify the same TRBV-TRBD-TRBJ gene rearrangements, hence further normalizing the different samples. PCR amplification of the TRBV-TRBD-TRBJ gene rearrangements, library construction and next generation sequencing (NGS) were performed as previously described (23). A paired-end sequencing protocol was followed in order to achieve double coverage in the TRB complementarity-determining region 3 (TRB CDR3) for each amplicon, thus increasing the accuracy of the results (for methodological details, see **Supplementary Methods**).

Paired-end read merging and strict length/quality filtering was performed by a purpose-built bioinformatics pipeline, as previously described; details are provided in **Supplementary Methods** (17, 24). Annotation of the TRBV-TRBD-TRBJ gene rearrangements was carried out by the IMGT/HighV-QUEST tool (25). Meta-data analysis was performed using the T cell Receptor/Immunoglobulin Profiler (TRIP) tool (26).

For the interpretation of the NGS results, the term “clonotypes” as used in this study refers to TRBV-TRBD-TRBJ gene rearrangements with unique pairs of TRBV genes and identical TRB CDR3 amino acid (aa) sequences within a sample. For the computation of clonotypes, we assessed only productive TRBV-TRBD-TRBJ gene rearrangements. Rearrangements carrying TRBV genes with <95% germline identity were discarded, being considered as sequences with unacceptable error rate. Details of the overall metrics regarding the NGS data are given in **Supplementary Table S2**.

The 10 clonotypes with the highest frequency within a sample are herein referred to as “major”. The relative frequency of each clonotype/sample was calculated as the number of TRBV-TRBD-TRBJ gene rearrangements corresponding to this particular clonotype divided by the total number of productive, filtered-in TRBV-TRBD-TRBJ gene rearrangements of the sample.

To overcome empirical assumptions regarding the fraction of clonal expansions that was represented at a meaningful frequency, hence potentially claiming biological relevance, “significantly expanded clonotypes” were further defined based on a data-driven statistical analysis. The frequency distribution of clonotypes from all samples were transformed to z-scores; clonotypes with z-scores greater that 2 were selected. The minimum frequency of those clonotypes was set as the per sample threshold. The median value of all individual thresholds led to the identification of 0.216% as the discerning frequency above which a given clonotype would be considered as significantly expanded (27). A table listing the number of significantly expanded clonotypes per sample in each group is provided in **Supplementary Table S3**.

### Repertoire diversity and clonality

In order to characterize the complexity of the repertoire in each group, two different metrics were estimated: (i) repertoire diversity, describing the number of the different clonotypes present in each sample, and (ii) repertoire clonality, referring to skewed clone size distribution in a given sample or group of samples.

Regarding the former, repertoire diversity was described by Hill numbers (^1^D) using Recon for the calculations (28, 29). This algorithm is based on a modified maximum-likelihood method that calculates diversity not only by counting the different number of clonotypes identified per sample but also by evaluating the clonotype-size distribution, and further reconstructs the overall diversity of the initial samples. To assess the sensitivity of the calculated diversity value comparing the rare versus the abundant clonotypes, ^1^D numbers were considered for the analysis, meaning that each clonotype was exactly weighted by its proportional abundance (30). Through this approach, sampling biases, experimental errors, as well as the variable number of T cells that were captured in different blood samples can be normalized (29). Moreover, comparability of the results between different samples was reinforced through utilizing identical numbers of isolated cells as starting material for nucleic acid isolation, identical quantity of genomic DNA or cDNA for PCR amplification of TRBV-TRBD-TRBJ gene rearrangements, and similar number of sequencing reads for downstream bioinformatics analysis.

Regarding the assessment of repertoire clonality, the following values were determined: (i) the cumulative frequency (CF-10) of the major (top-10) clonotypes within a given sample; (ii) the median CF-10 value of all the samples within a given group (MCF-10) (17); (ii) the cumulative frequency of the significantly expanded clonotypes (CFEx) per sample; and (iv) the median CFEx value of all the samples within a given group (ex-MCF).

### TRBV gene repertoire

In order to evaluate the relative frequency of each TRB gene that partakes in the clonotype formation, clonotypes rather than individual TRBV-TRBD-TRBJ gene rearrangements were considered. In more detail, TRBV gene frequencies within a sample were calculated as the number of clonotypes using particular TRBV genes over the total number of clonotypes. For comparisons of the TRBV gene repertoire between the significantly expanded clonotypes and the remaining polyclonal background, an approach similar to differential expression analysis was implemented using *limma*, an R/Bioconductor software package (31).

### Clonotype comparisons

Extensive comparisons of the clonotypes between patients within a given group have been undertaken in order to identify common clonotypes in patients sharing the same genomic aberration (“group-specific” clonotypes). Furthermore, in order to identify common clonotypes regardless of the background of genomic aberrations (“public” clonotypes) comparisons between patients in different groups have been also pursued. Finally, cross comparisons have been performed against: (i) a well-annotated database of TR clonotypes with known antigen specificities (n=47,107 TR clonotypes), and (ii) TR clonotypes from our published NGS studies in monoclonal B-cell lymphocytosis (a potential precursor state to CLL), chronic idiopathic neutropenia and benign ethnic neutropenia (n=1,033,310) (32–34).

### Prediction of neoepitopes and search for neoepitope-specific clonotypes

In the case of *NOTCH1* and *TP53* mutations, each mutant nucleotide sequence was translated *in silico* into a protein sequence that was used as input for the TAP 1.0 tool (35). Based on the prediction model implemented by the tool, each examined epitope was characterized either as a tumor or non-tumor antigen with a given probability score. This score was estimated by TAP 1.0 based on the selected datasets of human tumor and non-tumor antigens that were used for the development and training of the tool (35).

For the prediction of possible neoepitopes deriving from the mutant p53 and Notch1 proteins in samples of the studied cohort, a region of 15aa length stretching in both sides of each particular mutated position was dissected into k-mers whose immunogenicity was calculated by TAP 1.0. In cases where an insertion/deletion occurred and led to a frameshift, the complete altered aa sequence downstream of this position was used for epitope prediction. All k-mers that were characterized as tumor antigens by TAP 1.0 with high probability score (over 0.9 in order to ensure stringency), were selected (‘filtered-in’) for further analysis. The lists of the filtered-in tumor-derived epitopes for each case are given in **Supplementary Table S4**.

Each group of possible tumor epitopes produced from a particular sample was used as input for ERGO-II (pEptide tcR matchinG predictiOn), a highly specific and generic TR-peptide binding predictor, allowing the *in silico* identification of specific TR clonotypes with high affinity for these epitopes (36). To that purpose, all the TR clonotypes of a given sample were checked for their binding affinity against all the respective filtered-in tumor-derived epitopes. The complete list of the MHC alleles (**Supplementary Table S5**) expressed in each sample was used as an additional feature in ERGO-II in order to refine the predicted interactions. Pairs of clonotypes and epitopes - MHC alleles with high binding affinity greater than 0.8, were further analyzed.

### HLA genotyping

Typing of the HLA-A, -B, -C (low resolution) and -DRB1 (allelic level high resolution determination) loci was performed by reverse PCR-SSOP (sequence specific oligonucleotide probe) using a commercially available kit (LABtype ^®^ RSSO, One Lambda, Thermo Fisher, CA, USA).

### Statistical analysis

Descriptive statistics (median, mean, min, max) were computed to characterize gene counts and clonotype frequency distribution. The non-parametric Kruskal Wallis and Mann–Whitney tests were used in order to identify differences between the studied variables at the distinct groups, while ANOVA was applied to compare diversity distributions amongst the different groups. Correction for multiple comparisons was obtained using the Benjamini-Hochberg *post hoc* test and the significance level was set at α = 0.05. All statistical analyses were performed with the statistical Package GraphPad Prism version 8 (GraphPad Software, Inc., San Diego, CA, USA) and R version 4.1.3.

## Results

### Distinct TR gene repertoire profiles in different groups of patients with CLL with distinct genomic aberrations

A total of 12,607,219 sequences were obtained by the sequencing experiments (median: 293,122/sample). After strict quality filtering, synthesis of the paired-end sequencing reads and annotation with the IMGT/HighV-QUEST tool, we finally evaluated 8,989,297 high-quality sequences corresponding to productive TRBV-TRBD-TRBJ rearrangements (median: 185,420/sample). Overall, we identified 465,401 distinct clonotypes with a median of 10,608 clonotypes/sample (**Supplementary Table S2**).

In general, cases carrying CNAs tended to display less diverse TR repertoires than cases carrying mutations in the *NOTCH1* and *TP53* genes, though not reaching statistical significance (α=0.06) likely due to the relatively small numbers of cases in each group. In more detail, the average Hill diversity numbers (^1^D) per group were 850, 1301 and 636 for cases carrying isolated del(11q), del(13q) or trisomy 12, respectively, versus 2051 and 1917 for cases carrying isolated *NOTCH1 or TP53* mutations, respectively. (**Supplementary Figure 1**).

### Prominent oligoclonal expansions in cases with copy number aberrations

**Figure 1:**
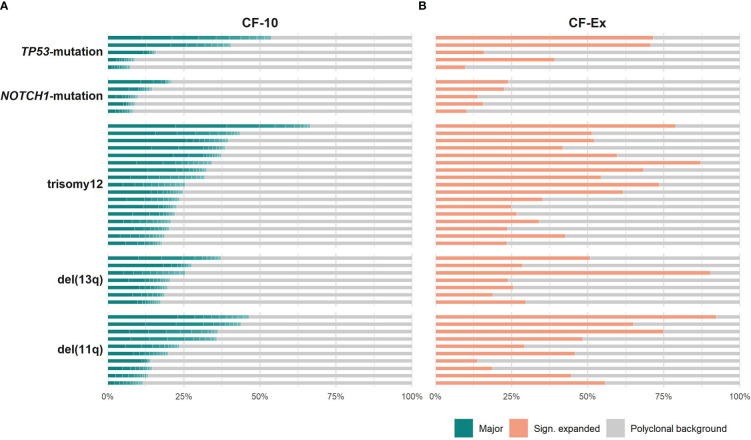
Clonality profiles of individual cases with CLL bearing distinct genomic aberrations. **(A)** Frequency (%) of the major clonotypes per sample of a given group. **(B)** Cumulative frequency (%) of the significantly expanded clonotypes per sample of a given group. Colored tiles depict each of the 10 major clonotypes within a sample and the fraction occupied by the significantly expanded clonotypes, respectively; the remaining clonotypes (polyclonal background) are depicted in grey.

All groups of the present study displayed repertoire restriction characterized by oligoclonal expansions. More precisely, the cumulative frequency of the major clonotypes (CF-10) ranged between 11.5-46.3, 17.3-37.1, 17.7-66.5 for cases carrying isolated del(11q), del(13q) or trisomy 12, respectively, versus 8.4-20.9 and 7.4-53.7 for cases carrying isolated NOTCH1 or TP53 mutations, respectively (**Figure 1A**). We detected similar patterns when assessing the expanded clonotypes (range: 5-31 expanded clonotypes/sample; median: 17 expanded clonotypes/sample) (**Figure 1B**).

**Figure 2:**
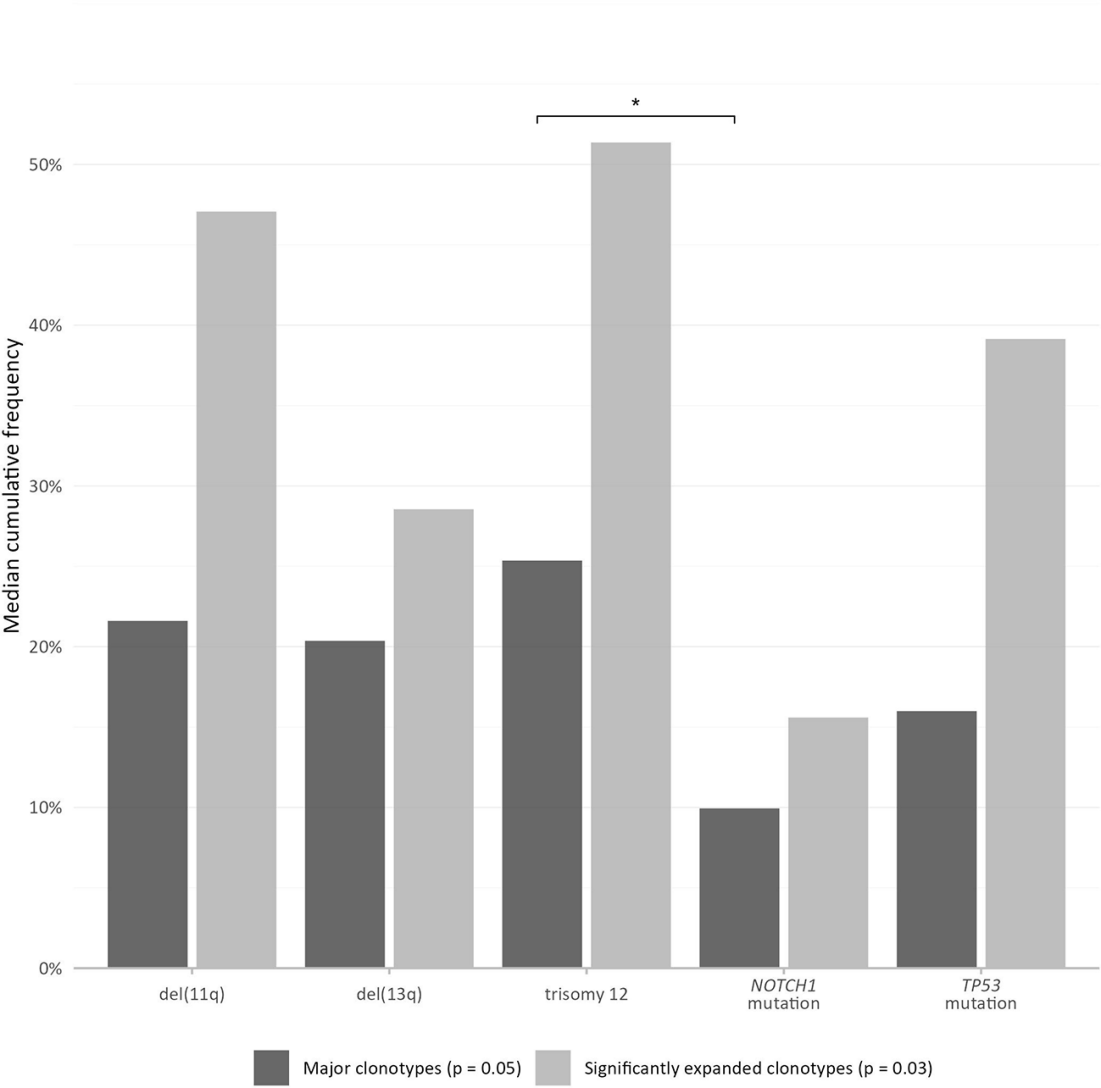
More pronounced TR repertoire skewing in cases bearing copy number aberrations, particularly trisomy 12, versus gene mutations. The bars represent the median cumulative frequency of the major clonotypes in each group (MCF-10) in dark grey color, and the median cumulative frequency of the significantly expanded clonotypes in each group (ex-MCF), in light grey color. The asterisk (*) refers to statistical significance at the level of α=0.03 following *post-hoc* comparisons using Benjamini-Hochberg correction.

The only difference concerned the *TP53*-mutation group, where the median cumulative frequency of the expanded clonotypes (MCF-Ex) was larger compared to the del(13q) group (**Figure 2**). The Kruskal-Wallis test documented a significant difference in the clonality profiles of the various groups of our study. More particularly, cases bearing CNAs displayed a significantly more oligoclonal repertoire compared to cases carrying *TP53* or *NOTCH1* mutations, irrespective of which repertoire fraction was tested (MCF-10, α=0.05; ex-MCF: α=0.03). Interestingly, *post-hoc* comparisons using Benjamini-Hochberg correction revealed that significant statistical differences (α=0.03) held mainly for the MCF-10 values of the trisomy 12 versus the *NOTCH1* mutation group (**Figure 2**).

### Distinct TRBV gene repertoires in groups of patients with CLL with distinct genomic aberrations

**Figure 3:**
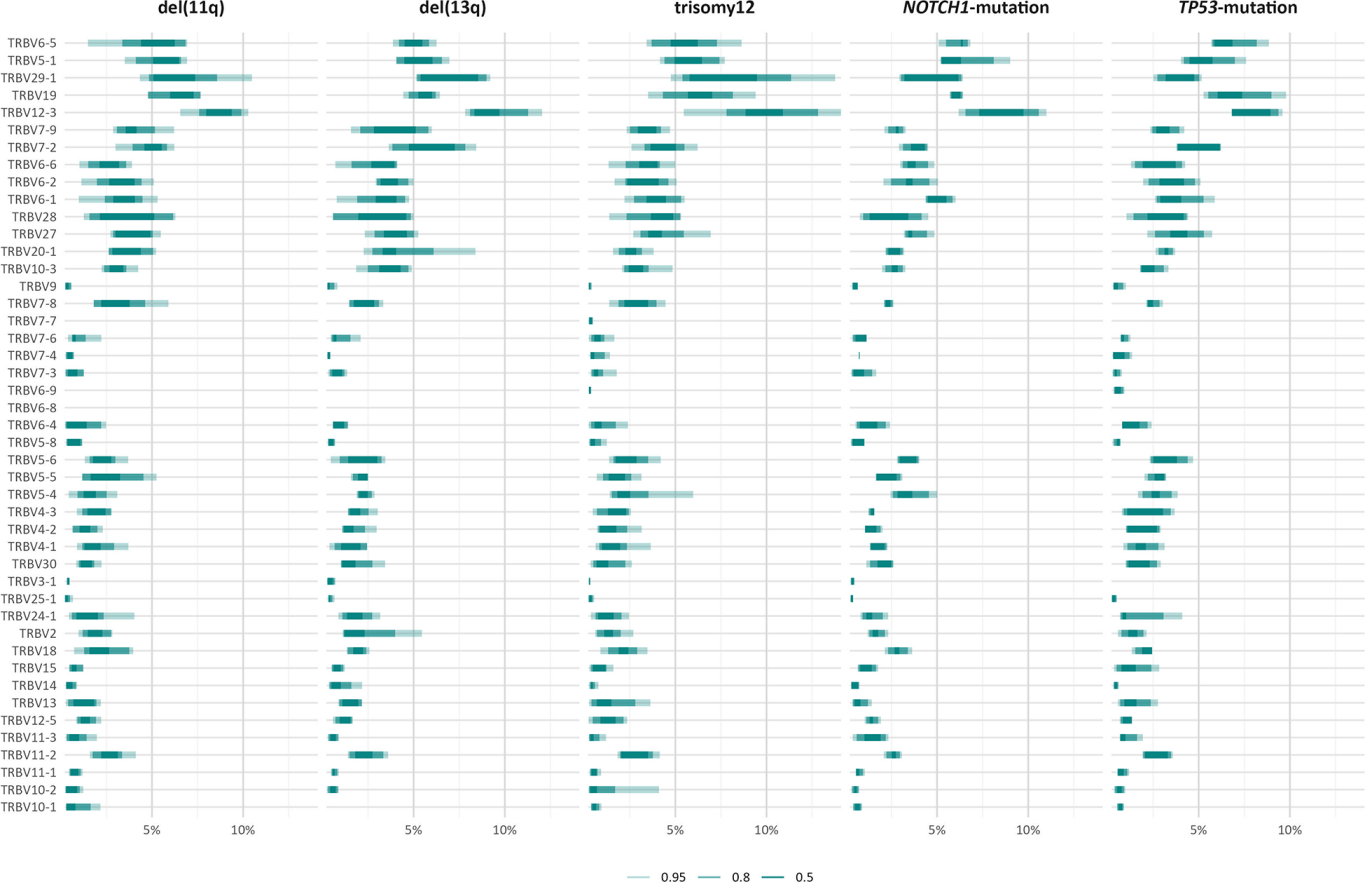
TRBV gene usage in different groups of patients with CLL carrying distinct genomic aberrations. The area including the frequency values of 50% of all observations in a given group is depicted in darker shade.

The TRBV gene repertoires of all groups were restricted (**Figure 3**). In more detail, TRBV12-3, TRBV29-1, TRBV19, TRBV5-1 and TRBV6-5 accounted for one-third of the total repertoire in all groups, except the *TP53*-mutation group, where the TRBV29-1 gene was under-represented (α=0.002) and, in contrast, the TRBV11-3 and TRBV6-9 genes were over-represented (α=0.01).

**Figure 4:**
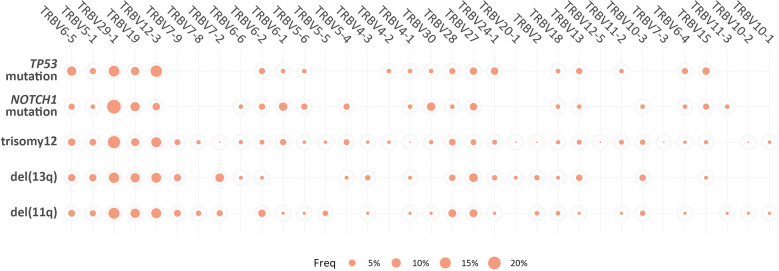
Biased TRBV gene usage in the major clonotypes in groups of patients with CLL bearing distinct genomic aberrations. The size of each bubble is proportional to the mean frequency (%) of each TRBV gene in the repertoire of the major clonotypes of all patients within a given group.

However, when we focused only on the major (top-10) clonotypes, differences in the TRBV gene rank became more obvious: TRBV29-1 was the most frequent TRBV gene in the major clonotypes of all groups, except for the *TP53*-mutation group, where TRBV12-3 predominated. In general, cases carrying *NOTCH1* and *TP53* mutations demonstrated more prominent, ‘group-biased’ differences in the usage of particular TRBV genes in the repertoire of major clonotypes (α<0.05) (**Figure 4**).

**Figure 5:**
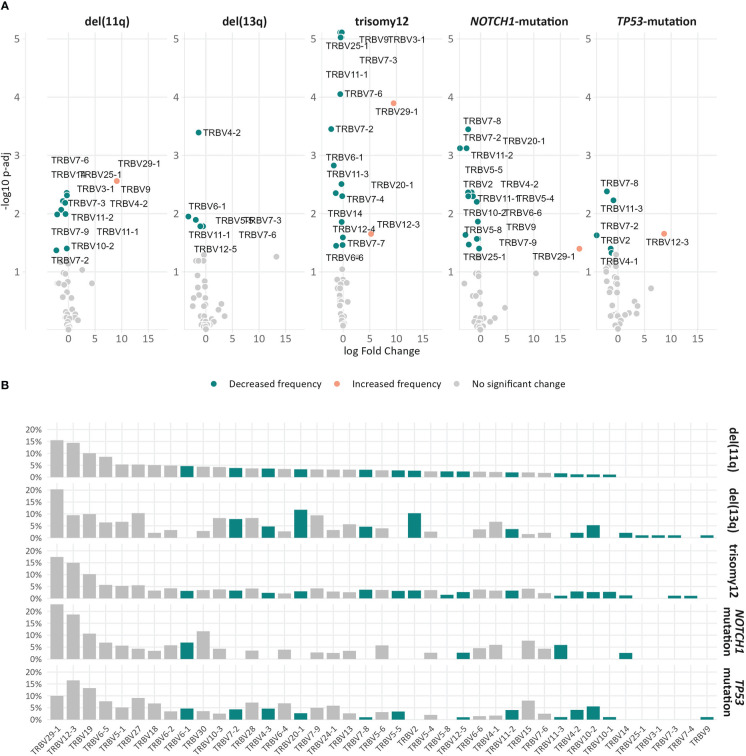
Differential usage of TRBV genes in the repertoires of the expanded clonotypes of the various groups in the study. **(A)** Volcano plots depict the significant variation in the TRBV gene usage in the repertoire of expanded clonotypes’ compared to the remaining polyclonal background within a given group. TRBV genes with decreased frequency in the repertoire of expanded clonotypes are depicted as petrol bubbles, whereas TRBV genes with increased frequency are depicted as orange bubbles. **(B)** TRBV gene frequencies in the repertoire of expanded clonotypes in each group. The colored bars represent TRBV genes with statistically significant differences between the different groups.

Skewing was also noted in the TRBV gene repertoire of the significantly expanded clonotypes in different groups. In more detail, TRBV29-1 appeared with increased frequency (α<0.05) in the repertoire of the significantly expanded clonotypes in the del(11q), trisomy 12 and *NOTCH1-*mutation groups when compared to the remaining polyclonal background (**Figure 5A**). On the other hand, the significantly expanded clonotypes of the *TP53-*mutation group displayed increased frequency in TRBV12-3 (α=0.02) (**Figure 5A**). In addition, differential expression analysis highlighted several genes in each group that were significantly depressed in the repertoire of the significantly expanded clonotypes versus the remaining clonotypes (**Figure 5A** and **Supplementary Table S6**).

Finally, comparisons of the repertoires of the significantly expanded clonotypes in the different groups highlighted asymmetric usage of certain TRBV genes (**Figure 5B**). In more detail, TRBV3-1 and TRBV25-1 were found exclusively in cases carrying del(13q) (α<0.0002). Additionally, TRBV2 was found exclusively in cases bearing CNAs, while TRBV7-4 prevailed only in cases with trisomy 12 (α<0.0003).

### Clonotype sharing: “Public” and “group-specific”

Comparisons of the clonotype repertoires of the various groups identified a total of 1,252 “public” clonotypes (0.26% of all analyzed clonotypes) that were shared by 2-7 samples of the present cohort regardless the background of genomic aberrations. Cross-comparisons against TRB gene rearrangement sequences from various other entities documented that none of these “public” clonotypes had been previously described in any other context, thus, they could be deemed as “CLL-biased”. Interestingly, patients of a given group also displayed a number of exclusive shared clonotypes (“group-specific”). In more detail, we identified 321 clonotypes exclusively shared between cases with del(11q), 55 in the del(13q) group, 180 in the trisomy 12 group, and, finally, 70 and 56 clonotypes in the *NOTCH1-* and *TP53*-mutant groups, respectively.

### *In silico* prediction reveals putative neoepitopes derived from *NOTCH1* and *TP53* gene mutations in CLL

The search for putative specific epitopes is far more straightforward in cases bearing point mutations or small insertions/deletions compared to cases with large genomic aberrations, since the latter involve many genes and affect many different pathways. On these grounds, we focused on the *NOTCH1*-mutation and *TP53-*mutation groups in order to explore the hypothesis that mutations in these genes could lead to the expression of neoepitopes that might select specific TR clones.

**Figure 6:**
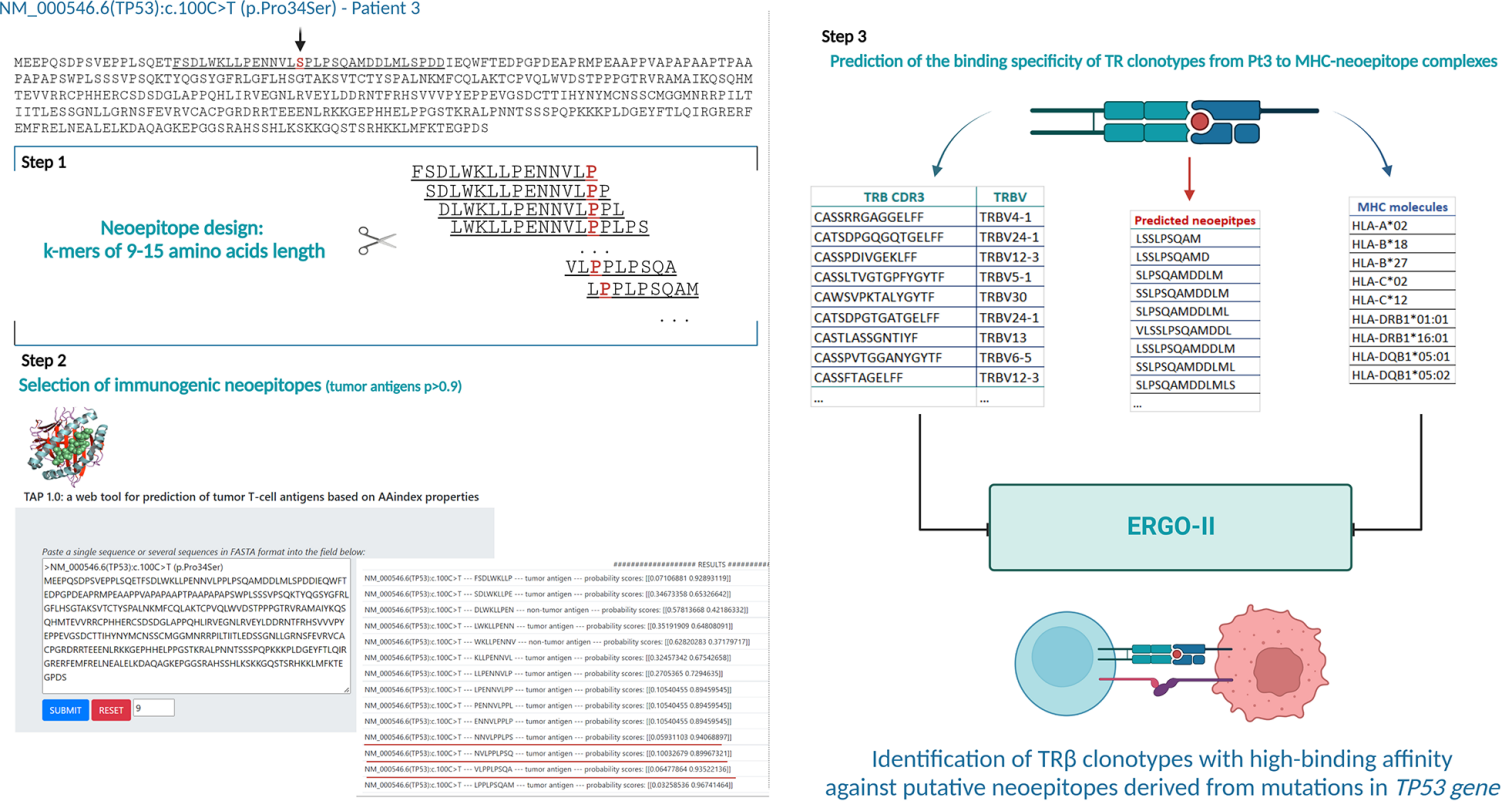
Analytical workflow for the prediction of neoepitopes and search for neoepitope-specific clonotypes (Illustrative case of Pt3). Identification of putative tumor-derived epitopes on TAP 1.0 tool using the mutant protein sequence of each case (Step 1 and 2). Lists of the TR clonotypes, *in silico* predicted neoepitopes and MHC molecules from each patient used for prediction of their binding affinity in ERGO-II (Step 3). Created with BioRender.com.

To that purpose, the mutant amino acid sequences of the Notch1 and p53 proteins from cases in the respective groups were dissected into epitopes of different length (k-mers of 9-15 amino acids) containing the mutant positions and all the produced epitopes were checked for their immunogenicity as described in the Materials and Methods section. In cases where the mutation resulted to a frameshift, the entire abnormal part of the protein was used for T-cell class I epitope prediction (**Figure 6**) Interestingly, mutations in the *TP53* gene led to the identification of greater number of possibly immunogenic neoepitopes, in sharp contrast to the cases with a 2-base pair frameshift deletion in *NOTCH1*: 1-182 predicted putative neoepitopes (median: 20 neoepitopes) in cases with mutations in *TP53* versus 3 predicted immunogenic neoepitopes derived from the 2-base pair deletion in *NOTCH1* (**Supplementary Table S4**). All examined cases are listed in **Table 1**, which also gives information about the identified mutations, their postulated molecular consequences and the number of filtered-in putative tumor-derived neoepitopes.

**Table 1:** Putative neoepitopes and predicted neoepitope-specific TR clonotypes in cases with *TP53* or *NOTCH1* mutations.

Case ID	Gene	Variant name	Molecular consequence	Structural motif	No of putative neoepitopes	No of neoepitope specific TR clonotypes	Percentage (%) of neoepitope specific TR clonotypes
**Pt3**	*TP53*	NM_000546.6: c.100C>T (p.Pro34Ser)	Substitution - Missense	N-term/ Transactivation	19	789	9.21
**Pt14**	*TP53*	- NM_000546.6: c.733G>A (p.Gly245Ser); - NM_000546.6: c.1146del (p.Lys382fs)	Substitution - Missense; Deletion /Insertion - Frameshift	L2/L3 | C - terminal	182	6,427	26.79
**Pt21**	*TP53*	NM_000546.6: c.464C>A (p.Thr155Asn)	Substitution - Missense	NDBL/beta-sheets	28	766	6.36
**Pt22**	*TP53*	NM_000546.6: c.607G>C (p.Val203Leu)	Substitution - Missense	NDBL/beta-sheets	1	42	1.19
**Pt23**	*NOTCH1*	NM_017617.5: c.7541_7542del (p.Pro2514fs)	Deletion - Frameshift	C-terminal heterodimerization and PEST domains	3	31	0.31
**Pt24**	*NOTCH1*	NM_017617.5: c.7541_7542del (p.Pro2514fs)	Deletion - Frameshift	C-terminal heterodimerization and PEST domains	3	124	1.64
**Pt26**	*TP53*	NM_000546.6: c.721T>C (p.Ser241Pro)	Substitution - Missense	L2/L3	20	194	3.24
**Pt27**	*NOTCH1*	NM_017617.5: c.7541_7542del (p.Pro2514fs)	Deletion - Frameshift	C-terminal heterodimerization and PEST domains	3	69	0.70

TR clonotypes from each of these cases along with the respective MHC alleles and the predicted putative neoepitopes were used as an input for the ERGO-II tool in order to predict their binding affinity. A different fraction of the repertoire (0.31-26.79% of all identified clonotypes) in each case corresponded to TR clonotypes identified by ERGO-II as displaying high binding affinity against putative neoepitopes derived from a particular genomic lesion (**Table 1**). Such postulated neoepitope-specific clonotypes ranged significantly in size from immunodominant to singletons i.e. clonotypes called out from a single sequencing read. Interestingly though, the most immunodominant clonotype (clonotype frequency: 11.1%) of Pt4 was characterized as neoepitope-specific displaying great binding affinity (score=0.85) against a 10-aa mutant epitope of p53 bound to HLA-B*27 based on ERGO-II. TR clonotypes identified by ERGO-II as high-affinity against putative neoepitopes were cross-compared against the complete clonotype repertoires of cases in the *TP53*-mutation and *NOTCH1-*mutation groups. This comparison did not identify identical clonotypes and/or identical CDR3s shared between the examined cases that belong to the same group. Interestingly, within the *NOTCH1*-mutation group, despite the fact that the examined cases bore the same 2-base pair frameshift deletion leading to same predicted neoepitopes, only 2 putative ‘epitope-specific’ TR clonotypes (TRBV12-3 - CASSLKSAGRNNSPLHF | TRBV29-1 - CSVVATVSGNTIYF) were shared between 2 of 3 examined cases. Moreover, no statistically significant differences were identified regarding the CDR3 length distribution and TRBV gene repertoire of the predicted neoepitope-specific TR clonotypes versus the remaining clonotypes of either the *TP53* or the *NOTCH1* mutant groups. Particularly regarding TRBV gene repertoire, we noted that the predicted neoepitope-specific TR clonotypes displayed the same group-specific restrictions (as documented above), with TRBV genes retaining almost the same rank in the repertoire when gene frequencies were considered.

## Discussion

The advent of NGS coupled with robust bioinformatics tools have revolutionized the field of immunogenetics towards precision medicine approaches. High-throughput studies allow for in-depth characterization of clonal dynamics of complex repertoires in various clinical contexts, including cancer, offering important insight in the implicated processes and mechanisms (37). In CLL, NGS immunogenetics has documented repertoire restrictions consistent with antigenic drive in the TR repertoire of the bystander T cells, alluding to dynamic interactions operating within the TME that arguably shape clonal behavior (16, 37–39). However, the exact nature of the selecting antigens remains to be determined (17, 40).

In previous studies from our group, we have provided evidence that at least a fraction of T cells in patients with CLL may specifically recognize leukemia-associated antigens, with the clonotypic BcR IG expressed by the malignant cells emerging as a potential source of neoepitopes selecting T cells (17, 19, 24). In the present study, we extended our analyses and examined common recurrent genomic aberrations of CLL cells as another possible source of immunogenic neoepitopes for T cells. More precisely, we assessed by NGS the TR gene repertoire in groups of patients with CLL carrying as an isolated genomic aberration one of del(11q), del(13q), trisomy 12, *TP53* or *NOTCH1* gene mutations.

In keeping with the literature, all cases of the present study displayed an oligoclonal TR repertoire, regardless of the underlying genomic background (13, 14, 16, 17). However, cases bearing CNAs, particularly those with trisomy 12, presented with more pronounced repertoire skewing compared to cases bearing isolated *TP53* or *NOTCH1* mutations. On these grounds, we argue that the observed differences may be linked to the particular genomic profile of each group. The possible confounding effect of shared (stereotyped) BcR IG was excluded since cases within a given group expressed unique clonotypic BcR IG. Another possible confounding factor referring to the well-known aged-related TR repertoire restriction was minimized given that the studied groups were aged-balanced (41).

TRBV gene repertoires were biased in all groups, albeit with no major differences between groups, possibly excepting the *TP53*-mutation group. The herein reported overrepresentation of the TRBV12-3, TRBV29-1, TRBV19, TRBV5-1 and TRBV6-5 genes has been previously described in CLL and can be explained by naturally occurring convergence also observed in the TR gene repertoires from healthy T cells (16, 42). Nonetheless, significant differences between the groups emerged when the analysis was restricted to either the major clonotypes or the significantly expanded clonotypes. In this case, we noted asymmetric usage of certain TRBV genes, prompting us to speculate that the respective clones may represent the most biologically relevant ones, possibly expanding in response to CLL-derived neoepitopes.

A notable finding of the present study concerns the identification of ‘public’ clonotypes i.e. clonotypes shared by different patients, which, however, had not been previously described in other contexts beyond CLL: on these grounds, such clonotypes may be deemed as “CLL-biased”. Moreover, certain “public” clonotypes were exclusive to a given group of our study (“group-specific”), supporting the hypothesis of shared immunogenic neoepitopes originating from a particular genomic aberration.

To validate this hypothesis, peptides derived from mutant variants of p53 and Notch1 displayed by the patients of the respective groups, were used along with their MHC alleles for the *in silico* identification of neoepitope-specific TR clonotypes. Indeed, patients with *TP53* or *NOTCH1* mutations presented a fraction of TR clonotypes, reaching up to 26.8% of the total number of clonotypes per case, that were identified *in silico* to bind with high affinity to immunogenic neoepitopes predicted to arise from each respective mutation in each case. Additional TRBV gene repertoire analysis on these TR clonotypes revealed that the group-specific restrictions identified in the particular fractions of the total repertoire (i.e. major and significantly-expanded clonotypes) were retained also in the fraction of neoepitope-specific TR clonotypes.

Postulated high affinity, neoantigen-specific clonotypes were ranging in frequency from immunodominant to clonotypes corresponding to a single sequencing read. This further highlights the value of the great analytical depth achieved by NGS, while also providing novel information regarding the diversity and frequency of such clonotypes that could contribute to therapeutic benefit. Arguably, the significant repertoire restrictions described in cases bearing CNAs may arise from dosage changes: increase of gene expression levels progressively leads to cellular effects due to protein aggregation, proteotoxicity and stress responses, that further shape T cell-mediated immunity, as described elsewhere (43, 44). Tracking the abundance and the relative clonal size of these neoantigen-specific clonotypes in the lymphoid tissues will offer valuable insights into the clonal dynamics in the different anatomical sites hosting microenvironmental interactions of the T cells with the CLL and antigen-presenting cells.

The strict inclusion criteria applied in the present study regarding the presence of isolated genomic aberrations led to a limited number of patients in each group, which is unsurprising considering that CLL clones are often characterized by multiple aberrations. Despite this limitation, our findings support our starting hypothesis and represent the first evidence that abnormalities in gene expression as well as gene dosage alterations caused by recurrent genomic aberrations in CLL may actively shape the TR gene repertoire. Admittedly, the presented immunogenetic evidence would require future formal validation, whereby the herein reported library of predicted neoantigens could serve as input for further experimentation using peptide-MHC-I multimers aiming to detect neoantigen-specific T cells circulating on the blood that could be engaged in anti-tumor responses against CLL cells. Arguably, relevant knowledge can meaningfully contribute to increasing the efficacy of T cell-based immunotherapeutic approaches driven by the ability to select and guide immune recognition by CLL-specific T cells.

## Data availability statement

The data presented in the study are deposited in the ArrayExpress repository (https://www.ebi.ac.uk/arrayexpress/), accession number E-MTAB-12216.

## Ethics statement

The studies involving human participants were reviewed and approved by CERTH’s ethics committee: 21/03/2019 Approval document: ETH.COM-45. The patients/participants provided their written informed consent to participate in this study.

## Author contributions

EV, AC, KS, and PB conceived of the presented idea. EV planned and carried out the experiments, performed the analysis and wrote the manuscript. ES and GG contributed to the experiments. NP and GK contributed on the statistical analysis and the design of the figures. SJ, NS, PR, LS, CO, and LM provided samples for the study. All authors provided critical feedback and helped shape the manuscript.
