# Supplementary material for: T cell receptor gene repertoire profiles in subgroups of patients with chronic lymphocytic leukemia bearing distinct genomic aberrations
Source: Front Oncol. 2023 Feb 1;13:1097942. doi: 10.3389/fonc.2023.1097942 (PMC9929157; doi:10.3389/fonc.2023.1097942)
Supplement: Supplementary file 1 [file DataSheet_1.pdf]

Supplementary Material

1 Supplementary Data

Genomic background characterization

In order to minimize the possible confounding effects of multiple recurrent aberrations within each sample and to establish that the analyzed patients carried only one of the aberrations of interest, we had selected cases that had been previously examined through comprehensive genomic characterization (including FISH, SNP arrays, gene panels and WES).

In more details:

1. To detect gene mutations, a previously published custom Agilent HaloPlex High Sensitivity (HS) panel design was modified using the Agilent SureDesign software (Agilent Technologies, USA).(1) The custom probes were designed to target the coding exons or hotspot regions of 13 genes of interest in CLL (*ATM*, *BIRC3*, *BTK*, *EGR2*, *FBXW7*, *MYD88*, *NFKBIE*, *NOTCH1*, *PLCG2*, *POT1*, *SF3B1*, *TP53* and *XPO1*). Libraries were prepared following the manufacturer's instructions and paired-end sequencing (150 bp reads) was performed on an Illumina's platform NextSeq500 (Illumina, San Diego, CA).
2. To detect the 2-base pair frameshift deletion in *NOTCH1*, exon 34 was amplified by PCR and then sequenced by Sanger sequencing following a previously published protocol.(2) Similarly, exons 4-8 of *TP53* gene were amplified by PCR and Sanger sequenced, as published previously.(3)
3. Whole exome sequencing (WES) libraries were prepared using TruSeq Exome Library Prep kit (Illumina, San Diego, CA) by the manufacturer's instructions. Sequencing experiments were carried out on Illumina's platform NextSeq500 following a paired-end sequencing protocol (Illumina, San Diego, CA). WES analysis performed by an in-house established computational pipeline, employing baw, sambamba and samtools consecutively.
4. Recurrent genomic aberrations were investigated also using Affymetrix GeneChip® Mapping Nsp1-250K arrays (Gene Chip Mapping 500K Assay Manual [P/N 701930 Rev2.], Affymetrix Inc., Santa Clara, CA, USA) according to the standard protocol.(4)
5. Chromosome banding analysis (CBA) and interphase fluorescence in situ hybridization (FISH) with probes for the detection of deletions of 6q21, 11q22 (ATM), 13q14 (D13S25 and D13S319), 17p13 (TP53), trisomy 12 and IGH rearrangements were performed as previously described.(5) CBA results reported according to the International System for Human Cytogenomic Nomenclature.
6. Array CGH analyses were carried out using 4x180K microarray slides (Agilent Technologies, Santa Clara, CA). Preparation of the microarray sample assay was performed as recommended by the manufacturer. Images were analyzed using the DEVA v1.2.1 (Roche NimbleGen Inc.) and Nexus Copy Number 6.1 (Biodiscovery, Inc., El Segundo, CA). Aberrations were evaluated in each sample using BioDiscovery's Fast Adaptive States Segmentation Technique (FASST2) algorithm. Copy number changes smaller than 50 kb, as well as alterations covered by a known Copy Number Variation in the database of genomic variants, or alterations located in non-coding regions were not reported.(6)

Next-generation sequencing: library preparation, analysis and interpretation

Targeted amplification of the TRBV-TRBD-TRBJ rearrangements in all samples was performed using TCRB Gene Clonality Assay—Gel Detection (Invivoscribe, SanDiego, CA, USA) and the NGS libraries were prepared based on the NEBNext® Ultra™ II DNA Library Prep Kit for Illumina (New England Biolabs Ipswich, MA, USA), as previously described.(7) The NGS libraries were sequenced on the Illumina® MiSeq platform using the MiSeq® Reagent Kit v3 (Illumina, San Diego, CA, USA). A paired-end sequencing protocol was followed in order to achieve double coverage in the TRB complementarity-determining region 3 (TRB CDR3) for each amplicon, thus increasing the accuracy of the results.

The basic steps of the bioinformatics analysis on the results of the NGS experiments included: (i) the assessment of the raw sequencing reads, (ii) the merge of paired-end reads, (iii) the annotation of the TRBV-TRBD-TRBJ gene rearrangements, and (iv) the clonotype computation followed by the meta-data interpretation.

In more detail, using the standard Illumina signal-processing software implemented by default on the sequencing platform low-quality sequences and/or sequences with unacceptable high error rate were filter-out. On the same time, all indexed reads were assigned to samples. The paired-end reads acquired during the sequencing were further characterized by a purpose-built algorithm performing: (i) length and quality filtering of raw reads, (ii) merging of filtered-in paired reads via local alignment, and (iii) length and quality filtering of the final, full-length sequences that fulfilled all the previous criteria. No base calls of Q-score<30 were allowed in the 75-nucleotide stretch upstream of the GXG motif in FR4, thus further increasing the CDR3 sequencing reliability.(8) Sequence rearrangements meeting the aforementioned criteria annotated by the IMGT/HighV-QUEST tool (http://www.imgt.org) and the metadata were processed by the T cell Receptor/Immunoglobulin Profiler (TRIP) analytical toolbox designed for immunogenetics analysis.(9,10) The main steps of results interpretation by TRIP consisted of selection of TRBV-TRBD-TRBJ rearrangements based on their functionality, clonotype computation, TRBV/ TRBD/ TRBJ gene repertoires extraction and cross-sample comparisons.

2 Supplementary Tables and Figures

Supplementary Tables

Supplementary table 1: Demographic and clinicobiological characteristics of the study group. | WES: whole genome sequencing, SS: Sanger sequencing for *TP53* and *NOTCH1* genes, SNP: single nucleotide polymorphism, NA: Not available

					Method of detection of genomic aberrations					
**ID**	**Sex**	**IGHV gene**	**IGHV region Identity %**	**Genomic abberation**	**WES**	**Targeted panel**	**SS**	**FISH**	**Array-CGH**	**SNP arrays**
**Pt1**	M	IGHV4-59*01	100	trisomy 12	X		X	X		
**Pt2**	M	IGHV4-39*06	99.6	trisomy 12	X		X	X		
**Pt3**	M	IGHV3-33*01	100	TP53 mutation	X		X	X		
**Pt4**	M	IGHV4-34*02	91.93	del(13q)	X		X	X		
**Pt5**	F	IGHV1-69*01	100	del(11q)	X		X	X		
**Pt6**	M	IGHV4-59*02	87	del(13q)	X		X	X		
**Pt7**	M	IGHV4-34*01	92.98	del(13q)	X		X	X		
**Pt8**	F	IGHV4-34*02	97.16	del(13q)	X		X	X		
**Pt9**	M	IGHV1-24*01	98.9	del(11q)	X		X	X		
**Pt10**	M	IGHV1-69*01	100	trisomy 12	X		X	X		
**Pt11**	M	IGHV5-51*01	100	trisomy 12	X		X	X		
**Pt12**	M	IGHV7-4-1*02	100	trisomy 12	X		X	X		
**Pt13**	M	IGHV1-69*12	100	del(11q)	X		X	X		
**Pt14**	F	IGHV3-53*01	100	TP53 mutation		X		X		
**Pt15**	F	IGHV3-15*01	100	NOTCH1 mutation		X		X		
**Pt16**	F	IGHV4-34*01	100	NOTCH1 mutation		X		X		
**Pt17**	F	NA	NA	trisomy 12		X		X		
**Pt18**	M	NA	NA	trisomy 12		X		X		
**Pt19**	M	IGHV3-23*04	95.10	trisomy 12		X		X		
**Pt20**	F	IGHV1-69*01	100	trisomy 12		X		X	X	
**Pt21**	M	IGHV3-72*01	99.13	TP53 mutation		X		X	X	
**Pt22**	M	IGHV3-11*01	91.67	TP53 mutation		X		X	X	
**Pt23**	F	IGHV2-5*06	95.53	NOTCH1 mutation		X		X	X	
**Pt24**	F	IGHV3-7*01	91.74	NOTCH1 mutation		X		X	X	

					Method of detection of genomic aberrations					
**ID**	**Sex**	**IGHV gene**	**IGHV region Identity %**	**Genomic abberation**	**WES**	**Targeted panel**	**SS**	**FISH**	**Array-CGH**	**SNP arrays**
**Pt25**	F	IGHV3-23*01	93.33	trisomy 12		X		X	X	
**Pt26**	F	IGHV1-8*01	93.4	TP53 mutation		X		X	X	
**Pt27**	M	IGHV3-30*02	100	NOTCH1 mutation		X		X	X	
**Pt28**	M	IGHV3-30*03	91.51	del(13q)			X			X
**Pt29**	M	IGHV3-48*03	100	trisomy 12			X			X
**Pt30**	M	IGHV4-b	100	trisomy 12			X			X
**Pt31**	F	IGHV3-72*01	97.01	trisomy 12			X			X
**Pt32**	M	IGHV3-48*03	97.77	del(13q)			X			X
**Pt33**	M	IGHV4-34*01	99.56	del(11q)			X			X
**Pt34**	F	IGHV3-15*07	92.83	del(11q)			X			X
**Pt35**	F	IGHV3-9*01	93.01	del(13q)			X			X
**Pt36**	F	IGHV3-15*01	100	trisomy 12			X			X
**Pt37**	M	IGHV3-21*01	100	trisomy 12			X			X
**Pt38**	M	IGHV4-39*01	96.63	del(11q)			X			X
**Pt39**	F	IGHV3-30-3*01	100	del(11q)			X			X
**Pt40**	M	IGHV1-69*01	100	del(11q)			X			X
**Pt41**	M	IGHV1-69*01	100	trisomy 12			X			X
**Pt42**	F	IGHV3-11*01	100	trisomy 12			X			X
**Pt43**	F	IGHV3-30*18	97.64	del(11q)			X			X
**Pt44**	M	IGHV3-21*01	98.67	del(11q)			X			X

Supplementary table 2: Overall metrics of the NGS data.

Group	Median number of raw reads/sample	Median number of productive sequences/sample	Range	Median number of distinct clonotypes/sample	Range
**del(11q)**	299,237	220,553	124,930 - 261,880	7,831	2,545 - 21,815
**del(13q)**	255,988	181,574	33,493 - 255,256	11,056	3,419 - 18,753
**trisomy 12**	293,122	215,356	44,132 - 293,869	10,608	2,325 - 26,719
***NOTCH1* mutation**	274,370	222,138	131,040 - 225,940	10,132	7,578 - 21,725
***TP53* mutation**	300,490	183,371	115,829 - 256,024	8,567	3,530 - 23,986

Supplementary table 3: Significantly expanded clonotypes. The number of clonotypes per sample that presented with frequency above 0.216% and considered as significantly expanded.

Manuscript ID	Genomic aberration	No of expanded clonotypes (f>0.216%)
Pt1	trisomy 12	17
Pt2	trisomy 12	11
Pt3	TP53 mutation	17
Pt4	del(13q)	12
Pt5	del(11q)	28
Pt6	del(13q)	14
Pt7	del(13q)	10
Pt8	del(13q)	10
Pt9	del(11q)	5
Pt10	trisomy 12	31
Pt11	trisomy 12	20
Pt12	trisomy 12	21
Pt13	del(11q)	13
Pt14	TP53 mutation	10
Pt15	NOTCH1 mutation	12
Pt16	NOTCH1 mutation	9
Pt17	trisomy 12	16
Pt18	trisomy 12	17
Pt19	trisomy 12	19
Pt20	trisomy 12	15
Pt21	TP53 mutation	7
Pt22	TP53 mutation	26
Pt23	NOTCH1 mutation	14
Pt24	NOTCH1 mutation	17
Pt25	trisomy 12	13
Pt26	TP53 mutation	24
Pt27	NOTCH1 mutation	9
Pt28	del(13q)	5
Pt29	trisomy12	18
Pt30	trisomy12	20
Pt31	del(13q)	22
Pt32	del(13q)	27
Pt33	del(11q)	29
Pt34	del(13q)	23
Pt35	del(13q)	20
Pt36	trisomy12	19
Pt37	trisomy12	27
Pt38	del(11q)	15
Pt39	del(11q)	27
Pt40	del(11q)	22
Pt41	trisomy12	23
Pt42	trisomy12	13
Pt43	del(11q)	13
Pt44	del(11q)	25

Supplementary table 4: Tumor-derived epitopes for each case bearing *TP53* or *NOTCH1* mutations. Complete lists with the predicted tumor epitopes derived from a particular lesion on *TP53* or *NOTCH1*.

Case ID	Gene	Variant name	Molecular consequence	No of predicted neo-epitopes
**Pt3**	**TP53**	**NM_000546.6:c.100C>T p.Pro34Ser**	**Substitution - Missense**	**19**
LSSLPSQAM	VLSSLPSQAMDD	LSSLPSQAMDDLM	LSSLPSQAMDDLML	LSSLPSQAMDDLMLS
VLSSLPSQAM	SSLPSQAMDDLM	SSLPSQAMDDLML	SSLPSQAMDDLMLS	SSLPSQAMDDLMLSP
VLSSLPSQAMD	SLPSQAMDDLML	SLPSQAMDDLMLS	SLPSQAMDDLMLSP	SLPSQAMDDLMLSPD
SLPSQAMDDLM	VLSSLPSQAMDDL	VLSSLPSQAMDDLM	VLSSLPSQAMDDLML	
**Pt14**	**TP53**	**NM_000546.6:c.733G>A p.Gly245Ser**<br>**NM_000546.6:c.1146del p.Lys382fs**	**Substitution - Missense**<br>**Deletion/Insertion - Frameshift**	**182**
YNYMCNSSC	SKKGQSTSR	TSRHKKLMFK	CNSSCMGSMNRR	TSSSPQPKKKPLD
NYMCNSSCM	STSRHKKLM	SRHKKLMFKT	NSSCMGSMNRRP	SSSPQPKKKPLDG
YMCNSSCMG	SRHKKLMFK	YNYMCNSSCMG	VRVCACPGRDRR	RGRERFEMFRELN
MCNSSCMGS	RHKKLMFKT	NYMCNSSCMGS	GRDRRTEEENLR	KSKKGQSTSRHKK
CNSSCMGSM	YNYMCNSSCM	YMCNSSCMGSM	RDRRTEEENLRK	YMCNSSCMGSMNRR
SSCMGSMNR	NYMCNSSCMG	MCNSSCMGSMN	DRRTEEENLRKK	MCNSSCMGSMNRRP
SCMGSMNRR	YMCNSSCMGS	CNSSCMGSMNR	RRTEEENLRKKG	PGRDRRTEEENLRK
CMGSMNRRP	MCNSSCMGSM	NSSCMGSMNRR	RKKGEPHHELPP	GRDRRTEEENLRKK
MGSMNRRPI	CNSSCMGSMN	SSCMGSMNRRP	LPPGSTKRALPN	RDRRTEEENLRKKG
GSMNRRPIL	SSCMGSMNRR	NLLGRNSFEVR	PPGSTKRALPNN	LPPGSTKRALPNNT
SMNRRPILT	SCMGSMNRRP	SFEVRVCACPG	PGSTKRALPNNT	PPGSTKRALPNNTS
LGRNSFEVR	MGSMNRRPIL	FEVRVCACPGR	KRALPNNTSSSP	KRALPNNTSSSPQP
GRNSFEVRV	NSFEVRVCAC	EVRVCACPGRD	LPNNTSSSPQPK	RALPNNTSSSPQPK
SFEVRVCAC	SFEVRVCACP	VRVCACPGRDR	PNNTSSSPQPKK	ALPNNTSSSPQPKK
FEVRVCACP	FEVRVCACPG	RDRRTEEENLR	NNTSSSPQPKKK	LPNNTSSSPQPKKK
EVRVCACPG	EVRVCACPGR	RRTEEENLRKK	NTSSSPQPKKKP	PNNTSSSPQPKKKP
VRVCACPGR	VRVCACPGRD	KKGEPHHELPP	TSSSPQPKKKPL	NNTSSSPQPKKKPL
RVCACPGRD	RRTEEENLRK	LPPGSTKRALP	SSSPQPKKKPLD	NTSSSPQPKKKPLD
VCACPGRDR	NLRKKGEPHH	PPGSTKRALPN	SSPQPKKKPLDG	TSSSPQPKKKPLDG
RDRRTEEEN	PPGSTKRALP	PGSTKRALPNN	QIRGRERFEMFR	LKSKKGQSTSRHKK
RRTEEENLR	PGSTKRALPN	KRALPNNTSSS	RGRERFEMFREL	KSKKGQSTSRHKKL
NLRKKGEPH	GSTKRALPNN	PNNTSSSPQPK	KSKKGQSTSRHK	PGRDRRTEEENLRKK
RKKGEPHHE	STKRALPNNT	NNTSSSPQPKK	SKKGQSTSRHKK	GRDRRTEEENLRKKG
STKRALPNN	TKRALPNNTS	NTSSSPQPKKK	KKGQSTSRHKKL	PPGSTKRALPNNTSS
TKRALPNNT	KRALPNNTSS	TSSSPQPKKKP	YNYMCNSSCMGSM	TKRALPNNTSSSPQP
KRALPNNTS	PNNTSSSPQP	SSSPQPKKKPL	YMCNSSCMGSMNR	KRALPNNTSSSPQPK
NTSSSPQPK	NNTSSSPQPK	SSPQPKKKPLD	MCNSSCMGSMNRR	RALPNNTSSSPQPKK
TSSSPQPKK	NTSSSPQPKK	SPQPKKKPLDG	PGRDRRTEEENLR	ALPNNTSSSPQPKKK
SSSPQPKKK	TSSSPQPKKK	IRGRERFEMFR	GRDRRTEEENLRK	LPNNTSSSPQPKKKP
SSPQPKKKP	SSSPQPKKKP	RGRERFEMFRE	RDRRTEEENLRKK	PNNTSSSPQPKKKPL
SPQPKKKPL	SSPQPKKKPL	KSKKGQSTSRH	LPPGSTKRALPNN	NNTSSSPQPKKKPLD
PQPKKKPLD	SPQPKKKPLD	SKKGQSTSRHK	PPGSTKRALPNNT	NTSSSPQPKKKPLDG
TLQIRGRER	PQPKKKPLDG	KKGQSTSRHKK	TKRALPNNTSSSP	HLKSKKGQSTSRHKK
LQIRGRERF	RGRERFEMFR	YNYMCNSSCMGS	LPNNTSSSPQPKK	KSKKGQSTSRHKKLM
IRGRERFEM	KSKKGQSTSR	NYMCNSSCMGSM	PNNTSSSPQPKKK	
RGRERFEMF	KKGQSTSRHK	YMCNSSCMGSMN	NNTSSSPQPKKKP	
GRERFEMFR	KGQSTSRHKK	MCNSSCMGSMNR	NTSSSPQPKKKPL	
**Pt21**	**TP53**	**NM_000546.6:c.464C>A p.Thr155Asn**	**Substitution - Missense**	**28**
WVDSTPPPG	PPGNRVRAM	TPPPGNRVRA	TPPPGNRVRAM	CPVQLWVDSTPPPG
VDSTPPPGN	LWVDSTPPPG	PPPGNRVRAM	VQLWVDSTPPPG	PVQLWVDSTPPPGN
DSTPPPGNR	WVDSTPPPGN	QLWVDSTPPPG	VDSTPPPGNRVR	TCPVQLWVDSTPPPG
STPPPGNRV	VDSTPPPGNR	LWVDSTPPPGN	DSTPPPGNRVRA	CPVQLWVDSTPPPGN
TPPPGNRVR	DSTPPPGNRV	DSTPPPGNRVR	STPPPGNRVRAM	
PPPGNRVRA	STPPPGNRVR	STPPPGNRVRA	PVQLWVDSTPPPG	
**Pt22**	**TP53**	**NM_000546.6:c.607G>C p.Val203Leu**	**Substitution - Missense**	**1**
LIRVEGNLRL				
**Pt23**	**NOTCH1**	**NM_017617.5:c.7541_7542del p.Pro2514fs**	**Deletion - Frameshift**	**3**
PEHPFLTPS	PFLTPSRVP	PEHPFLTPSRVP		
**Pt24**	**NOTCH1**	**NM_017617.5:c.7541_7542del p.Pro2514fs**	**Deletion - Frameshift**	**3**
PEHPFLTPS	PFLTPSRVP	PEHPFLTPSRVP		
**Pt26**	**TP53**	**NM_000546.6:c.721T>C p.Ser241Pro**	**Substitution - Missense**	**20**
NYMCNSPCM	YNYMCNSPCM	CNSPCMGGMN	MCNSPCMGGMN	MCNSPCMGGMNR
YMCNSPCMG	NYMCNSPCMG	YNYMCNSPCMG	YNYMCNSPCMGG	YNYMCNSPCMGGM
MCNSPCMGG	YMCNSPCMGG	NYMCNSPCMGG	NYMCNSPCMGGM	YMCNSPCMGGMNR
CNSPCMGGM	MCNSPCMGGM	YMCNSPCMGGM	YMCNSPCMGGMN	MCNSPCMGGMNRR
**Pt27**	**NOTCH1**	**NM_017617.5:c.7541_7542del p.Pro2514fs**	**Deletion - Frameshift**	**3**
PEHPFLTPS	PFLTPSRVP	PEHPFLTPSRVP		

Supplementary table 5: MHC alleles expressed in each case. Typing of the HLA-A, -B, -C (low resolution) and -DRB1 (allelic level high resolution determination) loci was performed.

ID	MHC alleles									
**Pt3**	HLA-A*02		HLA-B*18	HLA-B*27	HLA-C*02	HLA-C*12	HLA-DR B1*01:01	HLA-DR B1*16:01	HLA-DQ B1*05:01	HLA-DQ B1*05:02
**Pt14**	HLA-A*11	HLA-A*24	HLA-B*07	HLA-B*15	HLA-C*03	HLA-C*07	HLA-DR B1*07	HLA-DR B1*15		
**Pt21**	HLA-A*01	HLA-A*11	HLA-B*08	HLA-B*35	HLA-C*04	HLA-C*07	HLA-DR B1*01:01	HLA-DR B1*03:01	HLA-DQ B1*02:01	HLA-DQ B1*05:01
**Pt22**	HLA-A*02	HLA-A*32	HLA-B*39	HLA-B*44	HLA-C*12	HLA-C*16	HLA-DR B1*07:01	HLA-DR B1*11:01	HLA-DQ B1*02:02	HLA-DQ B1*03:01
**Pt23**	HLA-A*01		HLA-B*08		HLA-C*07		HLA-DR B1*03:01		HLA-DQ B1*02:01	
**Pt24**	HLA-A*03	HLA-A*33	HLA-B*27	HLA-B*35	HLA-C*02	HLA-C*04	HLA-DR B1*03:01	HLA-DR B1*15:01	HLA-DQ B1*02:01	HLA-DQ B1*06:02
**Pt26**	HLA-A*01	HLA-A*02	HLA-B*08	HLA-B*44	HLA-C*07		HLA-DR B1*03:01	HLA-DR B1*11:01	HLA-DQ B1*02:01	HLA-DQ B1*03:01
**Pt27**	HLA-A*02	HLA-A*24	HLA-B*35	HLA-B*39	HLA-C*04	HLA-C*12	HLA-DR B1*07:01	HLA-DR B1*11:01	HLA-DQ B1*02:01	HLA-DQ B1*03:03

Supplementary table 6: TRBV gene repertoire. Differential TRBV gene usage in the expanded clonotype repertoire compared to the remaining polyclonal background | logFC: a log-fold change between TRBV gene frequency of the expanded clonotype repertoire and the frequency on the remaining polyclonal background; adj.P.Val: adj.P.Value is the p-value adjusted for multiple testing following Benjamini and Hochberg's method to control the false discovery rate.

del(11q)			
**Target**	**logFC**	**P.Value**	**adj.P.Val**
TRBV29-1	9.079555	5.85E-05	0.002749
TRBV7-6	-0.55498	0.00027	0.004389
TRBV14	-0.4564	0.000319	0.004389
TRBV3-1	-0.29326	0.000414	0.004389
TRBV25-1	-0.32633	0.000467	0.004389
TRBV9	-0.2901	0.000619	0.004852
TRBV4-2	-1.0444	0.000905	0.006079
TRBV7-3	-0.61974	0.001109	0.006514
TRBV11-2	-1.36396	0.001642	0.008575
TRBV11-1	-0.54874	0.002157	0.010138
TRBV7-9	-2.12495	0.002412	0.010304
TRBV10-2	-0.31498	0.010125	0.039655
TRBV7-2	-2.27468	0.011794	0.042641

Trisomy 12			
**Target**	**logFC**	**P.Value**	**adj.P.Val**
TRBV3-1	-0.23719	2.05E-07	7.74E-06
TRBV7-3	-0.5672	4.74E-07	7.74E-06
TRBV9	-0.26562	4.94E-07	7.74E-06
TRBV25-1	-0.31109	8.32E-07	9.44E-06
TRBV11-1	-0.48803	1.00E-06	9.44E-06
TRBV7-6	-0.55604	1.13E-05	8.87E-05
TRBV29-1	9.475572	1.89E-05	0.000127
TRBV7-2	-2.25933	6.00E-05	0.000353
TRBV6-1	-1.7745	0.000286	0.001493
TRBV11-3	-0.32985	0.000659	0.003098
TRBV20-1	-1.40655	0.001038	0.004434
TRBV7-4	-0.18559	0.001276	0.005
TRBV14	-0.28954	0.003838	0.013876
TRBV12-3	5.229178	0.006635	0.022274
TRBV12-4	-0.06542	0.008174	0.025612
TRBV7-7	-0.12072	0.011758	0.034538
TRBV6-6	-1.33366	0.012888	0.035631

del(13q)			
**Target**	**logFC**	**P.Value**	**adj.P.Val**
TRBV4-2	-1.35811	8.62E-06	0.000405
TRBV6-1	-3.28612	0.000476	0.011193
TRBV5-5	-1.88811	0.000815	0.012766
TRBV7-3	-0.64304	0.001446	0.015963
TRBV7-6	-0.5054	0.001698	0.015963
TRBV11-1	-0.51566	0.00241	0.016474
TRBV12-5	-1.03945	0.002454	0.016474

NOTCH1 mutation			
**Target**	**logFC**	**P.Value**	**adj.P.Val**
TRBV7-8	-2.33367	7.58E-06	0.000356
TRBV11-2	-2.63173	3.49E-05	0.000754
TRBV20-1	-2.63856	6.39E-05	0.000754
TRBV7-2	-3.87785	6.41E-05	0.000754
TRBV2	-1.68294	0.000488	0.00431
TRBV5-5	-2.35815	0.00055	0.00431
TRBV4-2	-1.43153	0.00083	0.005023
TRBV5-4	-2.31551	0.000855	0.005023
TRBV11-1	-0.72426	0.001198	0.006254
TRBV10-2	-0.5126	0.00291	0.013677
TRBV6-6	-2.84471	0.005417	0.023144
TRBV9	-0.4499	0.006881	0.026952
TRBV5-8	-0.70809	0.007495	0.027098
TRBV7-9	-2.2199	0.010113	0.033951
TRBV25-1	-0.28645	0.012688	0.039756
TRBV29-1	18.56844	0.013623	0.040018

TP53 mutation			
**Target**	**logFC**	**P.Value**	**adj.P.Val**
TRBV7-8	-2.08169	8.82E-05	0.004146
TRBV11-3	-0.8491	0.000251	0.005893
TRBV12-3	8.674279	0.001413	0.022133
TRBV7-2	-4.00013	0.002004	0.023552
TRBV2	-1.36689	0.004243	0.039883
TRBV4-1	-1.20962	0.006009	0.047067

Supplementary Figure

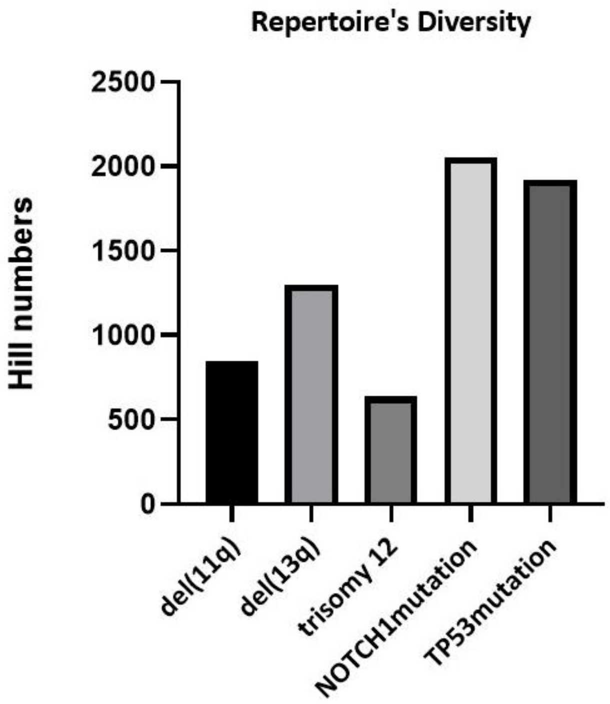

Supplementary figure 1: T cell receptor gene repertoire diversity expressed as Hill numbers ([1]D). Columns display the average value of Hill numbers for each group of the present study.

3 References

1. Agathangelidis A, Ljungström V, Scarfò L, Fazi C, Gounari M, Pandzic T, et al. Highly similar genomic landscapes in monoclonal B-cell lymphocytosis and ultra-stable chronic lymphocytic leukemia with low frequency of driver mutations. Haematologica. 2018 May 1;103(5):865 LP – 873.

2. Mansouri L, Cahill N, Gunnarsson R, Smedby KE, Tjönnfjord E, Hjalgrim H, et al. NOTCH1 and SF3B1 mutations can be added to the hierarchical prognostic classification in chronic lymphocytic leukemia. Leukemia. 2013 Feb;27(2):512–4.

3. Gunnarsson R, Isaksson A, Mansouri M, Göransson H, Jansson M, Cahill N, et al. Large but not small copy-number alterations correlate to high-risk genomic aberrations and survival in chronic lymphocytic leukemia: a high-resolution genomic screening of newly diagnosed patients. Leukemia 2010 24:1. 2009 Sep 10;24(1):211–5.

4. Gunnarsson R, Mansouri L, Isaksson A, Göransson H, Cahill N, Jansson M, et al. Array-based genomic screening at diagnosis and during follow-up in chronic lymphocytic leukemia. Haematologica [Internet]. 2011 Aug 1 [cited 2022 Sep 27];96(8):1161–9. Available from: https://haematologica.org/article/view/6043

5. Dicker F, Schnittger S, Haferlach T, Kern W, Schoch C. Immunostimulatory oligonucleotide-induced metaphase cytogenetics detect chromosomal aberrations in 80% of CLL patients: A study of 132 CLL cases with correlation to FISH, IgVH status, and CD38 expression. Blood. 2006 Nov 1;108(9):3152–60.

6. MacDonald JR, Ziman R, Yuen RKC, Feuk L, Scherer SW. The Database of Genomic Variants: a curated collection of structural variation in the human genome. Nucleic Acids Res. 2014 Jan 1;42(D1):D986–92.

7. Vlachonikola E, Vardi A, Stamatopoulos K, Hadzidimitriou A. High-Throughput Sequencing of the T-Cell Receptor Beta Chain Gene Repertoire in Chronic Lymphocytic Leukemia. In: Methods in Molecular Biology. Humana Press Inc.; 2019. p. 355–63.

8. Vardi A, Vlachonikola E, Karypidou M, Stalika E, Bikos V, Gemenetzi K, et al. Restrictions in the T-cell repertoire of chronic lymphocytic leukemia: High-throughput immunoprofiling supports selection by shared antigenic elements. Leukemia. 2017;31(7):1555–61.

9. Kotouza MT, Gemenetzi K, Galigalidou C, Vlachonikola E, Pechlivanis N, Agathangelidis A, et al. TRIP - T cell receptor/immunoglobulin profiler. BMC Bioinformatics. 2020 Sep 29;21(1):422.

10. Alamyar E, Duroux P, Lefranc MP, Giudicelli V. IMGT® Tools for the Nucleotide Analysis of Immunoglobulin (IG) and T Cell Receptor (TR) V-(D)-J Repertoires, Polymorphisms, and IG Mutations: IMGT/V-QUEST and IMGT/HighV-QUEST for NGS. In: Methods in molecular biology (Clifton, NJ). Methods Mol Biol; 2012. p. 569–604.
